# Potentially functional variants of *ERRFI1* in hypoxia‐related genes predict survival of non‐small cell lung cancer patients

**DOI:** 10.1002/cam4.70073

**Published:** 2024-08-03

**Authors:** Huilin Wang, Hongliang Liu, Guojun Lu, Xiaozhun Tang, Sheng Luo, Mulong Du, David C. Christiani, Qingyi Wei

**Affiliations:** ^1^ Department of Respiratory Oncology, Guangxi Cancer Hospital Guangxi Medical University Cancer Hospital Nanning Guangxi China; ^2^ Duke Cancer Institute, Duke University Medical Center Durham North Carolina USA; ^3^ Department of Population Health Sciences Duke University School of Medicine Durham North Carolina USA; ^4^ Department of Respiratory Medicine, Nanjing Chest Hospital, Affiliated Nanjing Brain Hospital Nanjing Medical University Nanjing Jiangsu China; ^5^ Department of Head and Neck Surgery, Guangxi Cancer Hospital Guangxi Medical University Cancer Hospital Nanning Guangxi China; ^6^ Department of Biostatistics and Bioinformatics Duke University School of Medicine Durham North Carolina USA; ^7^ Department of Environmental Health and Department of Epidemiology Harvard TH Chan School of Public Health Boston Massachusetts USA; ^8^ Department of Medicine Massachusetts General Hospital Boston Massachusetts USA; ^9^ Department of Medicine Duke University Medical Center Durham North Carolina USA; ^10^ Duke Global Health Institute, Duke University Medical Center Durham North Carolina USA

**Keywords:** *ERRFI1*, genome‐wide association study, hypoxia, non‐small cell lung cancer, overall survival, single‐nucleotide polymorphism

## Abstract

**Background:**

Hypoxia is often involved in tumor microenvironment, and the hypoxia‐induced signaling pathways play a key role in aggressive cancer phenotypes, including angiogenesis, immune evasion, and therapy resistance. However, it is unknown what role genetic variants in the hypoxia‐related genes play in survival of patients with non‐small cell lung cancer (NSCLC).

**Methods:**

We evaluated the associations between 16,092 single‐nucleotide polymorphisms (SNPs) in 182 hypoxia‐related genes and survival outcomes of NSCLC patients. Data from the Prostate, Lung, Colorectal, and Ovarian (PLCO) Cancer Screening Trial were used as the discovery dataset, and the Harvard Lung Cancer Susceptibility (HLCS) Study served as the replication dataset. We also performed additional linkage disequilibrium analysis and a stepwise multivariable Cox proportional hazards regression analysis in the PLCO dataset.

**Results:**

An independent SNP, *ERRFI1* rs28624 A > C, was identified with an adjusted hazards ratio (HR) of 1.31 (95% CI = 1.14–1.51, *p* = 0.0001) for overall survival (OS). In further analyses, unfavorable genotypes AC and CC, compared with the AA genotype, were associated a worse OS (HR = 1.20, 95% CI = 1.03–1.39, *p* = 0.014) and disease‐specific survival (HR = 1.21, 95% CI = 1.04–1.42, *p* = 0.016). Further expression quantitative trait loci analysis indicated that *ERRFI1* rs28624C genotypes were significantly associated with higher *ERRFI1* mRNA expression levels in the whole blood. Additional analysis showed that high *ERRFI1* mRNA expression levels were associated with a worse OS in patients with lung adenocarcinoma.

**Conclusion:**

Our findings suggest that genetic variants in the hypoxia‐related gene *ERRFI1* may modulate NSCLC survival, potentially through their effect on the gene expression.

## 1 INTRODUCTION

Lung cancer is a pervasive malignancy significantly impacting human health, particularly holding the highest global mortality rate among cancer‐related fatalities.^1^ In 2023, there were approximately 238,340 newly diagnosed cases of lung cancer in the United States, resulting in about 127,070 fatalities, underscoring its profound impact on population health.^2^ Non‐small cell lung cancer (NSCLC) constitutes approximately 85% of all lung cancer diagnoses. Despite notable progress in diagnosis and treatment, the prognosis for NSCLC remains persistently unfavorable.^3^ Identifying additional factors associated with the prognosis of NSCLC patients becomes imperative for clinically managing those patients with a bleak survival. Recently uncovered genetic variants, specifically single nucleotide polymorphisms (SNPs), have been demonstrated to be associated with survival of NSCLC patients.^4^, ^5^, ^6^ The discovery and characterization of functional SNPs not only enhance our comprehension of the genetic landscape but also present opportunities for translating this knowledge into more effective clinical management and therapeutic interventions.

Hypoxia, a condition characterized by insufficient oxygen supply to tissues, plays a pivotal role in the progression of various diseases, including cancer. It commonly occurs in solid tumors due to rapid cell proliferation, inadequate blood supply, and aberrant vasculature.^7^ In the presence of hypoxic pressure, a series of downstream pathways, including hypoxia‐inducible factor (HIF), autophagy, energy metabolic pathways, and cell stress pathways, are activated, facilitating cellular response to hypoxic stress.^8^, ^9^ This adaptive response promotes tumor cells to thrive in the hypoxic microenvironment, contributing to tumor growth and subsequent metastasis, such as seen in cancers of the colon, breast, prostate, pancreas, and lung.^10^, ^11^, ^12^, ^13^, ^14^ The tumor microenvironment in lung cancer often exhibits regions of hypoxia, and the hypoxia‐induced signaling pathways play a key role in the aggressive nature of lung cancer, affecting angiogenesis, immune evasion, and resistance to cancer therapies. Hypoxia increases vascular endothelial growth factor production, promoting the growth of blood vessels to sustain tumor growth.^15^ Targeting this process has become a therapeutic strategy in treating lung cancer.^16^, ^17^ Hypoxia in the tumor microenvironment can suppress the anti‐tumor immune response by promoting the recruitment of immunosuppressive cells and inhibiting the activity of cytotoxic T cells.^18^ This immune evasion contributes to the ability of lung cancer cells to escape immune surveillance, facilitating tumor progression. Understanding the intricate relationship between hypoxia and survival of patients with lung cancer can lead to the development of novel clinical management and therapeutic approaches.

In recent years, biological pathway analysis methods using genotyping data from genome‐wide association studies (GWAS) can reveal genetic variants within genes involved in cancer‐related pathways. To date, no published studies have explored the effects of genetic variants in the hypoxia‐related genes on survival of NSCLC patients. Therefore, we conducted the present study to investigate the associations between genetic variants in the hypoxia‐related genes and NSCLC survival, utilizing genotyping datasets from two GWAS studies previously published.

## 2 MATERIALS AND METHODS

### 2.1 Study populations

The discovery dataset was from the Prostate, Lung, Colorectal, and Ovarian (PLCO) Cancer Screening Trial, conducted by the National Cancer Institute (NCI). The PLCO trial involved 77,500 male and 77,500 female participants, aged 55–74 years, who were recruited from 10 medical centers in the United States between 1993 and 2011.^19^ Each participant was randomly assigned to either the intervention group receiving screening or the control group receiving the standard care. Baseline blood samples and demographic information were collected from all the participants. Over a 13‐year follow‐up period, meticulous records were maintained, covering the aspects of tumor diagnosis, histopathology, tumor staging, treatment modalities, and survival outcomes. Genotyping of whole blood genomic DNA was performed by using Illumina HumanHap240Sv1.0 and HumanHap550v3.0 platforms (dbGaP accession numbers: phs000093.v2.P2 and phs000336.v1.p1).^20^, ^21^ For survival analysis, a total of 1185 NSCLC patients of Caucasian ethnicity, with comprehensive personal information, were identified as eligible for inclusion in the present study. All the participants provided a written informed consent, having authorized the use of their datasets in the PLCO trial. Furthermore, the approval for the collection and use of data and samples was granted by the institutional review board at each participating institution.

The replication dataset was from the Harvard University Lung Cancer Susceptibility (HLCS) study, which was initiated in 1992.^22^ This dataset provided the needed information from 984 Caucasian patients with histologically confirmed NSCLC to validate the significant SNPs identified in the discovery dataset. In the HLCS study, DNA extraction from patient blood samples was conducted using the Auto Pure Large Sample Nucleic Acid Purification System by QIAGEN (Venlo, Limburg, Netherlands). Genotyping was executed using the Illumina Humanhap610‐Quad array, with subsequent imputation facilitated by the Minimac3 software, leveraging sequencing data from the 1000 Genomes Project.^23^

The approval for utilizing data from both datasets mentioned above, along with access to the dbGaP database (Project #6404), was granted by the Internal Review Board of Duke University School of Medicine (#Pro00054575) and the National Center for Biotechnology Information (NCBI). A detailed comparison between the PLCO trial (*n* = 1185) and the HLCS study (*n* = 984) is presented in Table S1.

### 2.2 Gene and SNP selection

The Hallmark gene sets are curated based on their ability to capture well‐defined biological states or processes, such as hypoxia, making them highly relevant for our study. We obtained the hypoxia‐related genes from the Hallmark gene sets within the Molecular Signatures Database (MSigDB) using the keyword “hypoxia.” To ensure the reliability of our analyses, we excluded 18 genes located on the X chromosome due to their unique inheritance patterns and potential for gender‐specific effects, which might introduce confounding variables. We identified 182 genes as candidate genes for further analyses (Table S2). Subsequently, we utilized Minimac4 for imputation on the Michigan Imputation Server (https://imputationserver.sph.umich.edu), employing the 1000 Genomes Project (phase 3) dataset of European ancestry as the reference panel. Next, we extracted all SNPs within ±2‐kb flanking regions of these genes, adhering to specific selection criteria, which included an *r* ^2^ value ≥0.3 (Figure S1), a minor allelic frequency (MAF) ≥0.05, a genotyping rate of ≥95%, and a Hardy–Weinberg equilibrium (HWE) *p*‐Value of ≥1 × 10^−5^. As a result, a total of 2225 genotyped SNPs were collected from the PLCO dataset, along with 13,867 imputed SNPs for subsequent analyses.

### 2.3 Statistical analysis

In the present study, the follow‐up duration for survival analysis was defined in the PLCO dataset as the period from the date of NSCLC diagnosis of the patients to the date of the last follow‐up or the date of death. The primary endpoint of the study was OS of the patients, with simultaneous analysis of disease‐specific survival (DSS) in a similar manner. Initially, a single‐locus analysis was conducted, followed by a multivariable Cox proportional hazards regression analysis. The Cox regression analysis involved adjustments for age, sex, smoking status, histopathology, tumor staging, chemotherapy, radiotherapy, surgery, and the first four principal components (Table S3), all under an additive genetic model. The objective was to assess the associations between each of 16,092 SNPs and survival of NSCLC patients in the discovery dataset. Considering that over 90% of SNPs were estimated to be in linkage disequilibrium (LD), a multiple testing correction was implemented using Bayesian false discovery probability (BFDP) with a cut‐off value of 0.80. This correction aimed to reduce the likelihood of potential false‐positive results as recommended.^24^ A prior probability of 0.10 was set to detect variant genotypes or minor allele‐associated hazards ratio (HR) with an upper limit of 3.0, and a significance level of *p* < 0.05 was considered.

We then replicated the identified SNPs from the discovery dataset using a multivariable Cox regression model in the replication dataset. The results from both datasets were subsequently combined through an inverse variance‐weighted meta‐analysis. Inter‐study heterogeneity was assessed using Cochran's *Q*‐test and the heterogeneity statistic (*I* ^2^) to guide the model selection. In cases without heterogeneity (*P*_het_ >0.100 and *I* ^2^ < 50%), we employed a fixed‐effects model; otherwise, a random‐effects model was used. To identify representative SNPs, we utilized online bioinformatics tools—specifically, RegulomeDB (http://www.regulomedb.org/) and HaploReg v4.2 (https://pubs.broadinstitute.org/mammals/haploreg/haploreg.php) with criteria such as *r* ^2^ < 0.8 and multifunctionality. In the pursuit of independent SNPs associated with NSCLC survival, we constructed a stepwise multivariable Cox regression model to incorporate the first four principal components of genotype data from the discovery dataset, along with 54 SNPs from prior publications in addition to the adjustments for available demographic and clinical variables. The outcomes for the selected SNPs were also visually presented through Manhattan plots by using Haploview v4.1 and regional association plots generated by using Locus Zoom (http://locuszoom.sph.umich.edu).

After adjusting for covariables in the discovery dataset with multiple comparison correction, we explored the associations of SNP genotypes with both NSCLC OS and DSS. Utilizing additive, dominant, and recessive models, we assessed survival‐associated SNP genotypes, categorizing those with an HR > 1 and a significance level of *p* < 0.05 as unfavorable genotypes. To assess the effects of all genotypes, particularly unfavorable ones, of the identified SNP on survival, we further generated Kaplan–Meier curves to visualize the effects on survival probabilities. Chi‐square Q‐tests were employed to explore interactions among various clinical subgroups in stratified analyses, allowing control for potential confounders and potential heterogeneity. Subsequently, using the “survival” and “time ROC” packages in R software (version 4.2.1), we constructed receiver operating characteristic (ROC) curves. The area under the curve (AUC) was calculated to develop survival prediction models, evaluating the predictive ability of combined genotypes for NSCLC survival.^25^ To confirm the independent association of SNP with the corresponding mRNA expression levels, an expression quantitative trait loci (eQTL) analysis was conducted, in which linear regression models were applied to mRNA expression data from 373 individuals of European descent in the 1000 Genomes Project as well as from 670 whole blood samples and 515 normal lung tissues in the genotype‐tissue expression (GTEx) project.^26^, ^27^

The analysis of mRNA expression levels in the Cancer Genome Atlas (TCGA) database included a comparison of 109 paired non‐small cell lung cancer tissues and adjacent normal tissues using the paired *t*‐test to explore differences in mRNA expression levels between tumor and adjacent normal tissues.^28^ The Gene Expression Profiling Interactive Analysis 2 (GEPIA2) online database (http://gepia2.cancer‐pku.cn) integrates published gene expression data and survival information from 960 NSCLC samples collected from TCGA databases.^29^ Kaplan–Meier (KM) analysis of this database was also used to visualize the association between mRNA expression and NSCLC survival. All statistical analyses were performed using SAS software (version 9.4; SAS Institute, Cary, NC, USA), unless otherwise specified.

## 3 RESULTS

### 3.1 Associations between SNPs in the hypoxia‐related genes and NSCLC survival

The characteristics of 1185 NSCLC patients from the discovery dataset and 984 NSCLC patients from the replication dataset are detailed in Table S1. After implementing multiple testing correction with BFDP ≤0.8, a total of 630 SNPs were found to be associated with NSCLC OS at a significance level of *p* < 0.05. These identified SNPs underwent further replication using the replication HLCS dataset, yielding 49 SNPs within seven genes, which remained statistically significant. Notably, three SNPs (*RORA* rs922782, *PLIN2* rs7867814, and *NEDDL4* rs1160748) had been previously reported in other distinct pathways.^4^, ^5^, ^6^ Among the seven genes, two (*PLIN2* and *PRKCA*) each featured only one associated SNP. Further LD analysis through Haploview software on the 47 SNPs resulted in eight SNPs from five genes as the tagger SNPs (Figure S2). Subsequently, these eight SNPs, along with the SNPs from *PLIN2* and *PRKCA*, totaling 10 SNPs, underwent further functional prediction and a stepwise multivariable Cox regression analysis (Table S4). Among these, six SNPs, in the presence of 54 previously published SNPs, were subjected to a stepwise multivariable Cox regression analysis.

Because the unavailability of detailed genotyping data in the HLCS dataset, we opted for a stepwise Cox regression analysis to account for clinical variables only available in the PLCO dataset, in assessing the independence of SNP effects on NSCLC survival, with adjustment for an additional 54 previously reported survival‐associated SNPs from the same dataset. Ultimately, among the unpublished SNPs, only rs28624 in *ERRFI1* maintained its independent association with NSCLC OS (Table 1). The meta‐analysis conducted on this *ERRFI1* rs28624 SNP across both discovery and replication datasets produced consistent results, illustrating uniformity between the discovery and replication datasets without heterogeneity (Table 2). Furthermore, a summary of the selected SNPs is presented in the Manhattan plot (Figure S3) and the regional association plot (Figure S4).

**TABLE 1:** The independent SNPs in multivariate Cox proportional hazards regression analysis with adjustment for other covariates and 54 previous published SNPs in the PLCO dataset.

Variables	Category	Frequency	HR (95% CI)^a^	*p* ^a^	HR (95% CI)^b^	*p* ^b^
Age	Continuous	1185	1.03 (1.01–1.04)	0.0002	1.04 (1.03–1.06)	<0.0001
Sex	Male	698	1.00		1.00	
	Female	487	0.78 (0.66–0.91)	0.002	0.65 (0.55–0.77)	<0.0001
Smoking status	Never	115	1.00		1.00	
	Current	647	1.72 (1.27–2.34)	0.0005	2.34 (1.70–3.24)	<0.0001
	Former	423	1.66 (1.24–2.21)	0.001	2.30 (1.69–3.12)	<0.0001
Histology	AD	577	1.00		1.00	
	SC	285	1.25 (1.03–1.52)	0.027	1.17 (0.95–1.44)	0.135
	Others	323	1.34 (1.12–1.601)	0.001	1.44 (1.19–1.75)	0.0002
Stage	I‐IIIA	655	1.00		1.00	
	IIIB‐IV	528	3.05 (2.49–3.74)	<0.0001	4.33 (3.47–5.41)	<0.0001
Chemotherapy	No	639	1.00		1.00	
	Yes	538	0.57 (0.47–0.68)	<0.0001	0.46 (0.38–0.56)	<0.0001
Radiotherapy	No	762	1.00		1.00	
	Yes	415	0.95 (0.80–1.13)	0.576	1.11 (0.93–1.33)	0.233
Surgery	No	637	1.00		1.00	
	Yes	540	0.20 (0.15–0.26)	<0.0001	0.17 (0.13–0.22)	<0.0001
*NEDDL4* rs11660748 A>G^c^	AA/AG/GG	937/229/19	1.15 (0.93–1.42)	0.197	1.36 (1.15–1.60)	0.0003
*PLIN2* rs7867814 G>A^c^	GG/GA/AA	908/246/21	1.30 (1.12–1.52)	0.001	1.12 (0.95–1.32)	0.169
*RORA* rs922782 T>G^c^	TT/TG/GG	333/602/250	0.77 (0.69–0.85)	<0.0001	0.85 (0.76–0.96)	0.006
*ERRFI1* rs28624 A>C	AA/AC/CC	766/366/42	1.24 (1.08–1.41)	0.002	1.31 (1.14–1.51)	0.0001

Abbreviations: CI, confidence interval; *ERRFI1*, ERBB receptor feedback inhibitor 1; HR, hazards ratio; *NEDDL4*, Neural Precursor Cell Expressed, Developmentally Down‐Regulated 4‐Like; PLCO, Prostate, Lung, Colorectal and Ovarian cancer screening trial; *PLIN2*, Perilipin 2; *RORA*, Retinoic Acid Receptor‐Related Orphan Receptor Alpha; SNP, single‐nucleotide polymorphisms.

^a^ Stepwise analysis included age, sex, smoking status, tumor stage, histology, chemotherapy, radiotherapy, surgery, PC1, PC2, PC3, PC4, and SNPs.

^b^ 54 published SNPs were used for post‐stepwise adjustment: rs779901, rs3806116, rs199731120, rs10794069, rs1732793, rs225390, rs3788142, rs73049469, rs35970494, rs225388, rs7553295, rs1279590, rs73534533, rs677844, rs4978754, rs1555195, **rs11660748 (*NEDDL4*)**, rs73440898, rs13040574, rs469783, rs36071574, rs7242481, rs1049493, rs1801701, rs35859010, rs1833970, rs254315, rs425904, rs35385129, rs4487030, rs60571065, rs13213007, rs115613985, rs9673682, rs2011404, **rs7867814 (*PLIN2*)**, rs2547235, rs4733124, rs11225211, rs11787670, rs67715745, **rs922782 (*RORA*)**, rs4150236, rs116454384, rs9384742, rs9825224, rs261083, rs76744140, rs6939623, rs113181986, rs2568847, rs11225211, rs10841847, rs2519996, rs36215.

^c^ Published SNPs.

**TABLE 2:** Associations of the independent SNP with overall survival in both discovery and validation datasets from two previously published NSCLC GWASs.

SNP	Allele^a^	Gene	FDR	BFDP	PLCO (*n* = 1185)			Harvard (*n* = 984)			Meta‐analysis		
					EAF	HR (95% CI)^b^	*p* ^b^	EAF	HR (95% CI)^c^	*p* ^c^	*P*_het_ ^d^ *I* ^2^	HR (95% CI)^e^	*p* ^e^
rs28624	A>C	ERRFI1	0.19	0.28	0.19	1.24 (1.09–1.41)	0.001	0.15	1.16 (1.00–1.34)	0.045	0.492 0	1.20 (1.09–1.32)	0.0002

Abbreviations: CI, confidence interval; EAF, effect allele frequency; HR, hazards ratio.

^a^ Reference > effect allele;

^b^ Adjusted for age, sex, stage, histology, smoking status, chemotherapy, radiotherapy, surgery, PC1, PC2, PC3, and PC4;

^c^ Adjusted for age, sex, stage, histology, smoking status, chemotherapy, radiotherapy, surgery, PC1, PC2, and PC3;

^d^ *P*_**het**_: *p* value for heterogeneity by Cochrane's *Q* test;

^e^ Meta‐analysis in the fix‐effect model.

**FIGURE 1:**
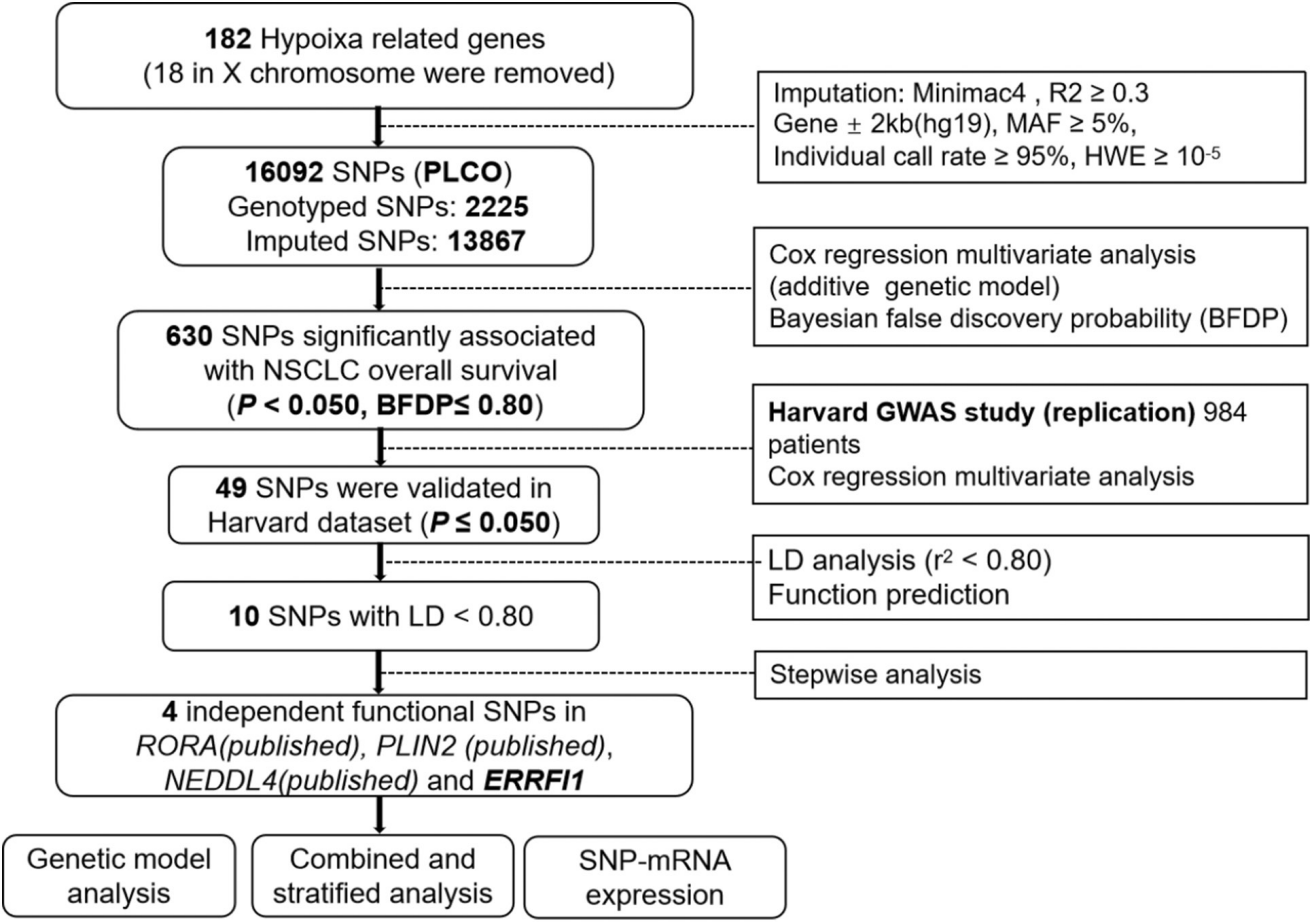
The flowchart of the present study. *ERRFI1*, ERBB Receptor Feedback Inhibitor 1; GWAS, Genome‐Wide Association Study; HLCS, Harvard lung cancer susceptibility study; *NEDDL4*, neural precursor cell expressed developmentally down‐regulated 4‐like; NSCLC, non‐small cell lung cancer; PLCO, Prostate, Lung, Colorectal and Ovarian cancer screening trial; *PLIN2*, perilipin 2; *RORA*, retinoic acid receptor‐related Orphan receptor alpha; SNP, single‐nucleotide polymorphism.

**FIGURE 2:**
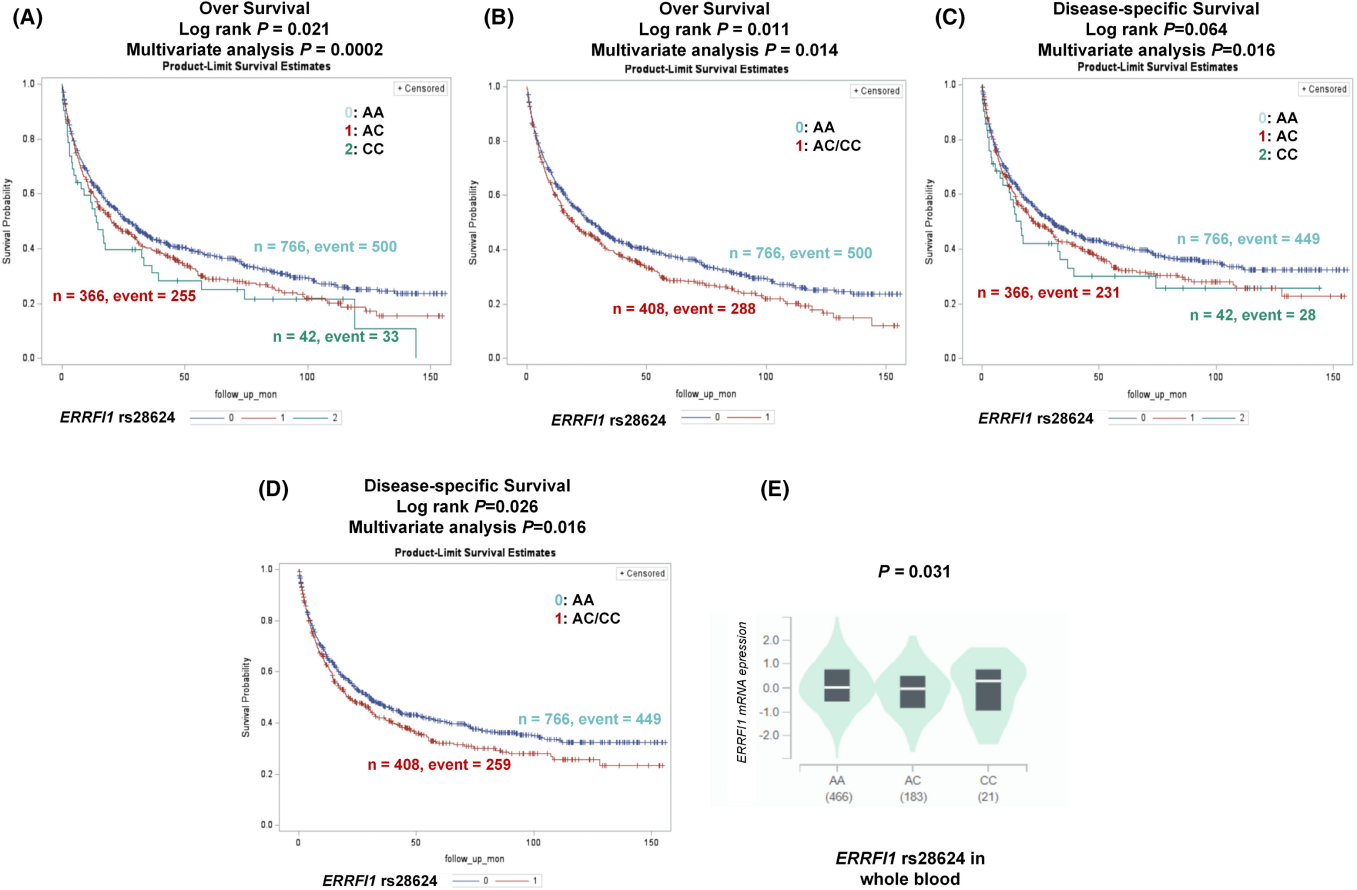
Prediction of survival with genotypes and eQTL analysis for SNP in *ERRFI1*. Kaplan–Meier survival curves for OS in the PLCO dataset for (A) each genotype and (B) the combined unfavorable genotypes; Kaplan–Meier survival curves for DSS in the PLCO dataset for (C) each genotype and (D) the combined unfavorable genotypes. (E) *ERRFI1* rs28624_C allele was associated with higher mRNA expression of *ERRFI1* in whole blood in GTEx project. eQTL, expression quantitative trait; *ERRFI1*, ERBB Receptor Feedback Inhibitor 1; PLCO, The Prostate, Lung, Colorectal and Ovarian Cancer Screening Trial; SNPs, single nucleotide polymorphisms. #Unfavorable genotypes were ERRFI1 rs28624 AC/CC.

As shown in Table 3, the carriers of the *ERRFI1* rs28624 C allele had an elevated risk of death or a poorer survival in NSCLC patients (*P*_trend_ = 0.006 for OS and *P*_trend_ = 0.019 for DSS); these findings are also illustrated in Kaplan–Meier survival curves (Figures 1 and 2A,C). In dominant genetic models, individuals with *ERRFI1* rs28624 AC + CC genotypes experienced significantly worse survival outcomes than those with the reference wildtype genotype (OS: HR = 1.20, 95% CI = 1.03–1.39, *p* = 0.014; DSS: HR = 1.21, 95% CI = 1.04–1.42, *p* = 0.016); these results are depicted in Kaplan–Meier survival curves (Figure 2B,D). In recessive genetic models, individuals with *ERRFI1* rs28624 CC genotypes, compared with those with AA+AC genotypes, had significantly worse survival outcomes (OS: HR = 1.97, 95% CI = 1.38–2.81, *p* = 0.0002; DSS: HR = 1.92, 95% CI = 1.31–2.82, *p* = 0.001).

**TABLE 3:** Associations of *ERRFI1* rs28624 A>C with survival of NSCLC in the PLCO study.

Genotype	Frequency	OS^a^			DSS^a^		
		Death (%)	HR (95% CI)	*p*	Death (%)	HR (95% CI)	*p*
***ERRFI1 rs28624 A>C*** ^b^							
AA	766	500 (65.27)	1.00		449 (58.62)	1.00	
AC	366	255 (69.67)	1.14 (0.98–1.33)	0.097	231 (63.11)	1.15 (0.98–1.36)	0.083
CC	42	33 (78.57)	2.05 (1.43–2.93)	<0.0001	28 (66.67)	2.01 (1.36–2.96)	0.0004
Trend test				0.0002			0.001
Dominant							
AA	766	500 (65.27)	1.00		449 (58.62)	1.00	
AC + CC	408	288 (70.59)	1.20 (1.03–1.39)	0.014	259 (63.48)	1.21 (1.04–1.42)	0.016
Recessive							
AA + AC	1132	755 (66.70)	1.00		680 (60.07)	1.00	
CC	42	33 (78.57)	1.97 (1.38–2.81)	0.0002	28 (66.67)	1.92 (1.31–2.82)	0.001

Abbreviations: CI, confidence interval; DSS, disease‐specific survival; HR, hazards ratio; NSCLC, non‐small cell lung cancer; OS, overall survival; PLCO, Prostate, Lung, Colorectal and Ovarian cancer screening trial; SNP, single nucleotide polymorphism.

^a^ Adjust sex, smoking status, histology, tumor stage, chemotherapy, surgery, radiotherapy, and principal components.

^b^ 11 missing date were ed for age, excluded.

### 3.2 Stratified analyses of independent SNP associated with NSCLC survival in the PLCO dataset

To enhance the precision of survival estimation, we classified *ERRFI1* rs28624 AC/CC as the unfavorable genotypes and conducted further stratified analyses using the PLCO dataset to investigate whether their effect on NSCLC survival was influenced by other factors such as age, sex, smoking status, histology, tumor stage, chemotherapy, radiotherapy, and surgery. The results indicated that neither the rs28624 AA group nor the rs28624 AC/CC unfavorable genotype displayed significant interactions with any demographic and clinical covariables in NSCLC OS and DSS (all *P*_inter_ > 0.05, Table S5).

### 3.3 Time dependent AUC and ROC curve for independent SNP for the prediction of NSCLC survival

Then, we assessed predictive capability of *ERRFI1* rs28624 for 5‐year NSCLC survival by contrasting the AUCs of models incorporating clinical variables with that integrating the identified independent SNP. The ROC curves illustrated that the inclusion of the identified SNP in the Cox model with 5‐year OS clinical variable borderline increased the AUC from 86.99% to 87.64% (*p* = 0.088, Figure S5B). Additionally, the incorporation of the identified SNP into the Cox model with 5‐year DSS prediction also borderline raised the AUC from 86.70% to 87.47% (*p* = 0.063, Figures S5A,C,D). These findings indicated that the identified SNP alone did not statistically significantly improve the predictive performance of the models for 5‐year survival.

### 3.4 The eQTL analysis

To perform the eQTL analysis, we started with the analysis of RNA‐Seq data derived from lymphoblastoid cell lines of 373 individuals of European descent participating in the 1000 Genomes Project. The results did not show a correlation between *ERRFI1* rs28624 C allele and its corresponding mRNA expression levels across all three genetic models (Figures S6A–C). Following this initial analysis, we also performed an eQTL analysis utilizing data obtained from 515 normal lung tissues and 670 whole blood samples sourced from the GTEx project. This analysis revealed a statistically significant association between the rs28624 C allele and elevated expression levels of *ERRFI1* in whole blood samples (*p* = 0.031, Figure 2E). Specifically, participants with the CC genotype had higher mRNA expression levels than those with AA and AC genotypes. However, it is noteworthy that such an association was not observed in normal lung tissues (*p* = 0.835, Figure S6D).

In analyzing functional relevance of the identified independent SNP, bioinformatics evaluations employing RegulomeDB and Haploreg revealed some functional implications associated with *ERRFI1* rs28624 A>C. Specifically, the variant influences enhancer histone marks and DNAse sensitivity and also induces alterations in sequence motifs, as detailed in Table S4.

### 3.5 Differential mRNA expression analysis and survival of NSCLC

**FIGURE 3:**
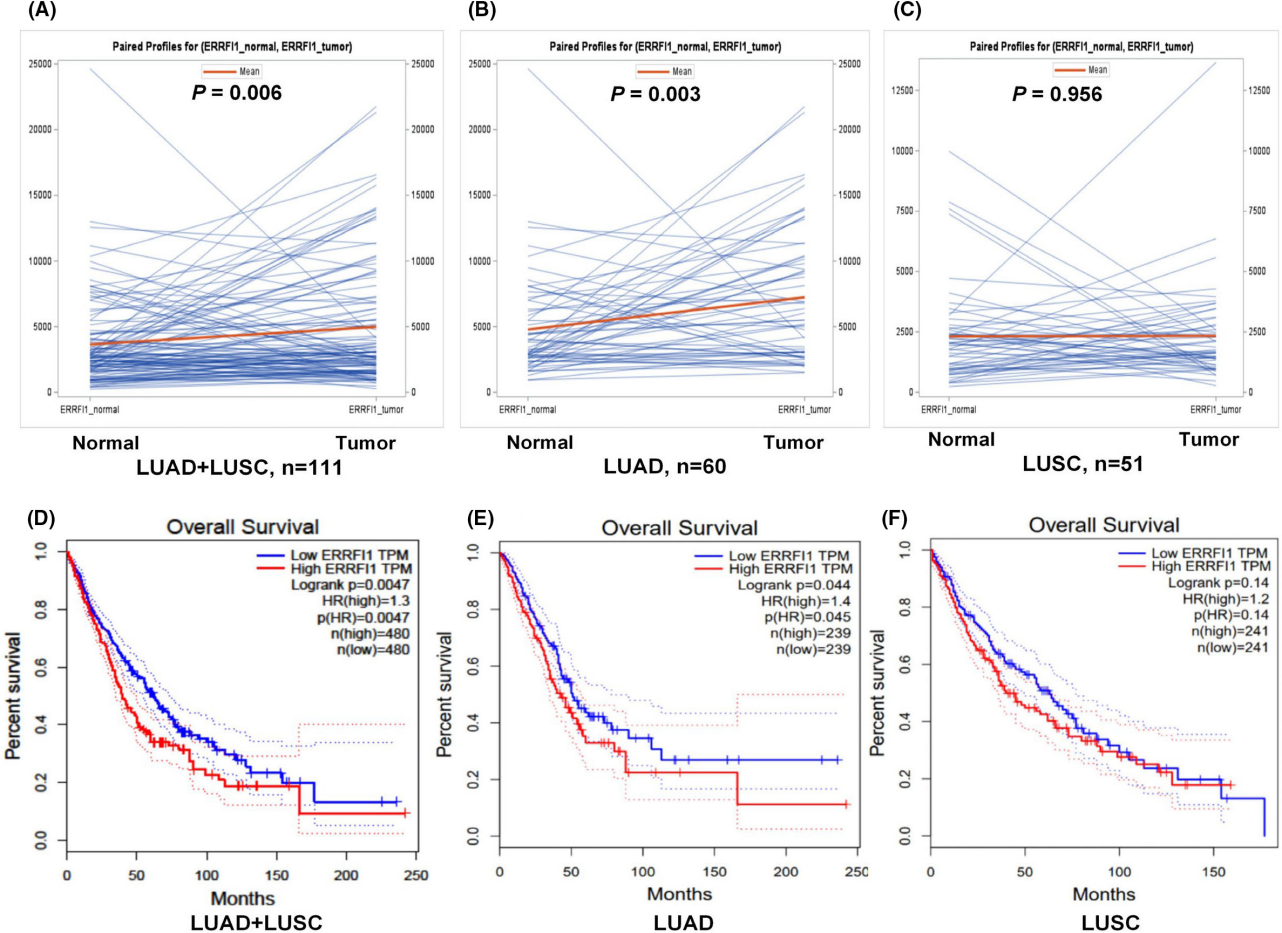
Differential mRNA expression analysis and survival of *ERRFI1* in the TCGA and GEPIA dataset. In TCGA database, relative to normal tissues, elevated *ERRFI1* mRNA levels were observed in (A) both overall LUAD and LUSC tissues, as well as in (B) isolated LUAD tissues, but no significant difference was observed in (C) LUSC tissues. In the GEPIA database, increased *ERRFI1* mRNA levels were correlated with worse survival outcomes in (D) both overall LUAD and LUSC patients, as well as in (E) isolated LUAD patients, but no significant difference in (F) LUSC patients. *ERRFI1*, ERBB Receptor Feedback Inhibitor 1; GEPIA, Gene Expression Profiling Interactive Analysis; LUAD, lung adenocarcinoma; LUSC, lung squamous cell carcinoma; TCGA, The Cancer Genome Atlas.

To further explore potential roles of the independent SNP in the survival of NSCLC, we first performed an analysis of *ERRFI1* mRNA expression levels sourced from the TCGA database, which included 109 pairs of tumor samples (58 lung adenocarcinoma and 51 lung squamous cell carcinoma) and their corresponding adjacent normal tissues. Subsequently, we evaluated the associations between gene mRNA expression levels and the survival of NSCLC patients utilizing the GEPIA2 database. As depicted in Figure 3A–C, *ERRFI1* mRNA expression levels were significantly elevated in the combined data for both lung adenocarcinoma (LUAD) and lung squamous cell carcinoma (LUSC) tissues (*p* = 0.006) as well as for LUAD tissues (*p* = 0.003), but not for LUSC tissues (*p* = 0.956). However, higher mRNA expression levels of *ERRFI1* were associated with a worse OS in the combined LUAD and LUSC group (*p* = 0.0047, Figure 3D), consistent with the findings in LUAD patients (*p* = 0.044, Figure 3E), but not in LUSC patients (*p* = 0.14, Figure 3F).

## 4 DISCUSSION

In the present study, we have tested the hypothesis that SNPs in the hypoxia‐related genes are associated with the survival of NSCLC using two publicly available GWAS databases. Through multivariable Cox analysis, we identified an independent SNP, *ERRFI1* rs28624 A>C, which was significantly associated with worse OS and DSS in American Caucasians. Subsequent eQTL analysis revealed a correlation between the rs28624 C variant allele and higher *ERRFI1* mRNA expression levels in whole blood samples sourced from the GTEx project. Moreover, *ERRFI1* mRNA expression levels significantly increased in paired LUAD tissues but not in paired LUSC tissue. The observed differences in *ERRFI1* mRNA levels between LUAD and LUSC may be attributed to the inherent biological heterogeneity characterizing these distinct subtypes of NSCLC. Consistently, higher mRNA expression levels of *ERRFI1* were associated with a poorer OS in patients with LUAD, while no significant association was observed in patients with LUSC. Overall, these findings suggest that genetic variants in the hypoxia‐related gene *ERRFI1* are associated with the survival of NSCLC, particularly in patients with LUAD, likely through alterations in the gene expression.

In our in silico analysis (http://www.mulinlab.org/vportal/apir.html?q=rs28624&g=hg19), the rs28624 variant allele (A/C) located at chr1:8084355 influences the binding affinity of several transcription factors (TFs), which may subsequently affect the expression of the *ERRFI1* gene. Figure S7B suggests that rs28624 may modulate gene expression by altering enhancer activity. Figure S8 further explores the regulatory impact of rs28624 by examining its effect on transcription factor binding motifs. The variant allele of rs28624 significantly affects the binding affinities of several transcription factors, as indicated by binding affinity decreases of USF2 (HM00936), ZZZ3 (HM07399), CTCF (HM02667), RAD21 (HM02636), MAX (HM00295), CTCF (HM01328), and NR3C1 (HM01174) and binding affinity increases of CEBPA (HM03470), SOX6 (HM06519), MEF2A (HM03380), SOX6 (HM06559), TBP (HM01158), and HNF4A (HM08596). Therefore, this variant influences the binding affinity of multiple transcription factors (TFs), which in turn may alter the expression of the *ERRFI1* gene.

The *ERRFI1* (ERBB receptor feedback inhibitor 1), also known as *MIG‐6* (mitogen‐inducible gene 6 protein), *Gene‐33*, and *RALT* (receptor‐associated late transducer), is situated on chromosome 1p36. It plays a crucial role in cellular signal transduction.^30^ As an early response gene, *ERRFI1* encodes a non‐kinase scaffold adaptor protein, with its expression induced by various stimuli, such as stress, growth factors, hormones, and hypoxia.^31^, ^32^, ^33^ Its primary function is the negative regulation of ERBB family receptors, including the epidermal growth factor receptor (EGFR) and other related receptors.^30^ *ERRFI1* has been implicated in regulating cellular processes, such as apoptosis, migration, and invasion, demonstrating a tumor‐suppressive role in glioblastoma, liver cancer, and endometrial cancer cells.^34^, ^35^, ^36^ However, its role in breast cancer progression appears to be context‐dependent, exhibiting both pro‐tumorigenic and anti‐tumorigenic effects in different types and stages of breast cancer progression.^37^, ^38^

However, *ERRFI1* possesses a dual mechanism for inhibiting the EGFR signaling, involving a direct binding to EGFR, leading to the inhibition of EGFR catalytic activity, as well as directing lysosomal degradation of EGFR.^39^, ^40^ EGFR‐tyrosine kinase Inhibitors (EGFR‐TKIs) have become crucial in the treatment of NSCLC with *EGFR* activating mutations, which are relatively rare in LUSC (approximately 4%).^41^ Adenocarcinoma, a predominant histological subtype of NSCLC, often features mutations in *EGFR*. In our analysis of the GEPIA2 data, high expression levels of *ERRFI1* were associated with a poor survival in patients with LUAC. However, in a mouse model of LUAC driven by mutant *EGFR*, the absence of ERRFI1 accelerates both the onset and progression of the tumour.^42^ In contrast, another preclinical study suggested that suppression *ERRFI1* overcomes acquired EGFR‐TKIs resistance in LUAD.^43^ Furthermore, patients with a lower ERRFI1/EGFR ratio exhibited elevated response rates to EGFR‐TKI, along with significantly extended progression‐free survival.^44^ In NSCLC cellular models, tumors with a low *ERRFI1*/miR200c ratio showed a higher sensitivity to EGFR‐TKI.^45^ Hypoxia‐induced upregulation of *ERRFI1* results in dormancy and resistance to EGFR‐TKI in primary cultured NSCLC cells with EGFR mutations.^45^ Overall, these results suggest that elevated ERRFI1 expression levels may be associated with suboptimal therapeutic efficacy in the EGFR‐TKI treatment. Clinical data also showed that patients with high *ERRFI1* expression levels had a poor prognosis after the EGFR‐TKI treatment in LUAC with the *EGFR* mutation.^45^ Similarly, another study suggested that high ERRFI1 expression levels were positively correlated with a poor prognosis and EGFR‐TKI resistance in patients with LUAC with *EGFR* mutation.^43^ Furthermore, Maity et al.^42^ demonstrated that although *ERRFI1* deficiency reduced mouse survival due to an accelerated tumorigenesis, mutant *EGFR* can partially circumvent inhibition by *ERRFI1* in LUAC cells through phosphorylation of ERRFI1 on a key residue. Under *EGFR* mutation conditions and prolonged exposure to EGFR‐TKIs, phosphorylated ERRFI1 ceases to operate as a tumor suppressor; instead, it actively promotes the survival of cancer cells.^43^

In summary, the present study is the first, to the best of our knowledge, to focus on the associations of potentially functional genetic variants in the hypoxia‐related genes with the survival of NSCLC patients. By utilizing two previously published GWAS datasets, we identified a potentially functional SNP in *ERRFI1*, which may serve as a biomarker for survival in NSCLC by a potential mechanism of modulating the expression of the gene. To validate these findings and to decipher the intricate molecular mechanisms involved, further experimental investigations are warranted. While the present study provides valuable insights, it is important to acknowledge its limitations. First, our analysis was based on GWAS datasets from populations of Caucasian descent, raising questions about the generalizability to other ethnic groups. Additionally, the two GWAS databases may inherently be constrained by limited availability of treatment details and EGFR mutation status, which hinder further in‐depth analysis. Moreover, although we identified a significant survival‐associated SNP, our study did not delve into the detailed functional consequences of this genetic variant at the molecular level. Lastly, our analysis was confined to the available data, potentially leading to the omission of other relevant confounding factors or interactions that may have played a role. Nevertheless, our study contributes to the understanding of the role that genetic variants of hypoxia‐related genes may play in NSCLC survival, offering potential insights for future investigations.

## AUTHOR CONTRIBUTIONS

**Qingyi Wei:** Conceptualization (lead); data curation (equal); formal analysis (equal); funding acquisition (lead); investigation (lead); methodology (lead); project administration (lead); resources (lead); software (lead); supervision (lead); validation (lead); writing – review and editing (equal). **Huilin Wang:** Conceptualization (equal); data curation (lead); formal analysis (lead); investigation (lead); methodology (equal); project administration (equal); writing – original draft (lead); writing – review and editing (equal). **Hongliang Liu:** Conceptualization (equal); data curation (equal); investigation (equal); methodology (lead); software (equal); supervision (equal); validation (equal); writing – review and editing (equal). **Guojun Lu:** Data curation (equal). **Xiaozhun Tang:** Data curation (equal). **Sheng Luo:** Funding acquisition (equal); writing – review and editing (equal). **Mulong Du:** Formal analysis (equal); resources (equal). **David C. Christiani:** Data curation (equal); funding acquisition (equal); investigation (equal); resources (equal); writing – review and editing (equal).

## FUNDING INFORMATION

None.

## CONFLICT OF INTEREST STATEMENT

The authors declare no conflict of interest.

## ETHICS STATEMENT

Each of the original studies with the approval by the Institutional Review Boards of the Participating institutions received written informed consent from the participants. Further ethical approval for the use of data and samples from the PLCO trial and HLCS, as well as access to the database of Genotypes and Phenotypes (dbGaP) under Project #6404, was provided by the Duke University School of Medicine's Internal Review Board (IRB), with approval number Pro00103470.

## Supporting information


Figures S1–S2.

Figures S3–S6.

Figures S7–S8.

Tables S1–S5.

## Data Availability

Only publicly available data were used in this study, and data sources and handling of these data are described in the Materials and Methods. The datasets used for the analyses described in the present study were obtained from dbGaP (http://www.ncbi.nlm.nih.gov/gap) through dbGaP accession number phs000336.v1.p1 and phs000093.v2.p2. Further details and other data that support the findings of this study are available from the corresponding author upon request.
