# Supplementary material for: Potentially functional variants of ERRFI1 in hypoxia‐related genes predict survival of non‐small cell lung cancer patients
Source: Cancer Med. 2024 Aug 3;13(15):e70073. doi: 10.1002/cam4.70073 (PMC11297539; doi:10.1002/cam4.70073)
Supplement: Supplementary file 1 — Figures S1–S2. [file CAM4-13-e70073-s004.doc]

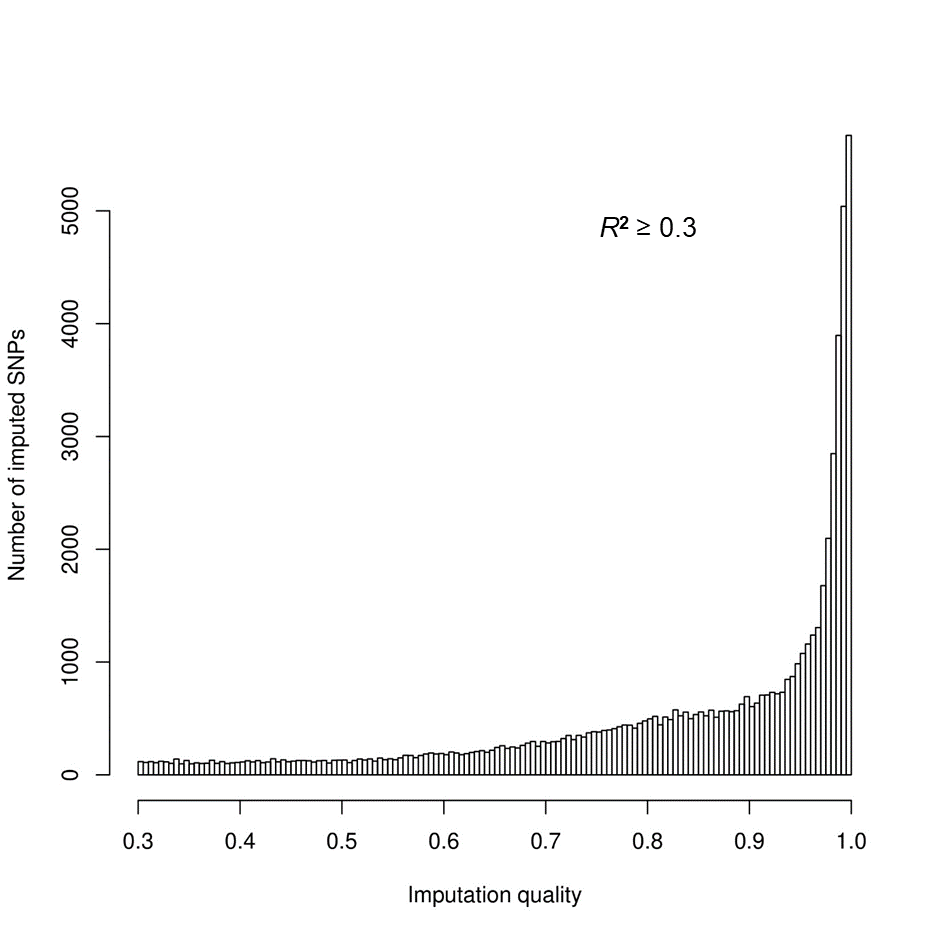


**SUPPLEMENTARY FIGURE 1:** The distribution of the imputation information score of the present study.


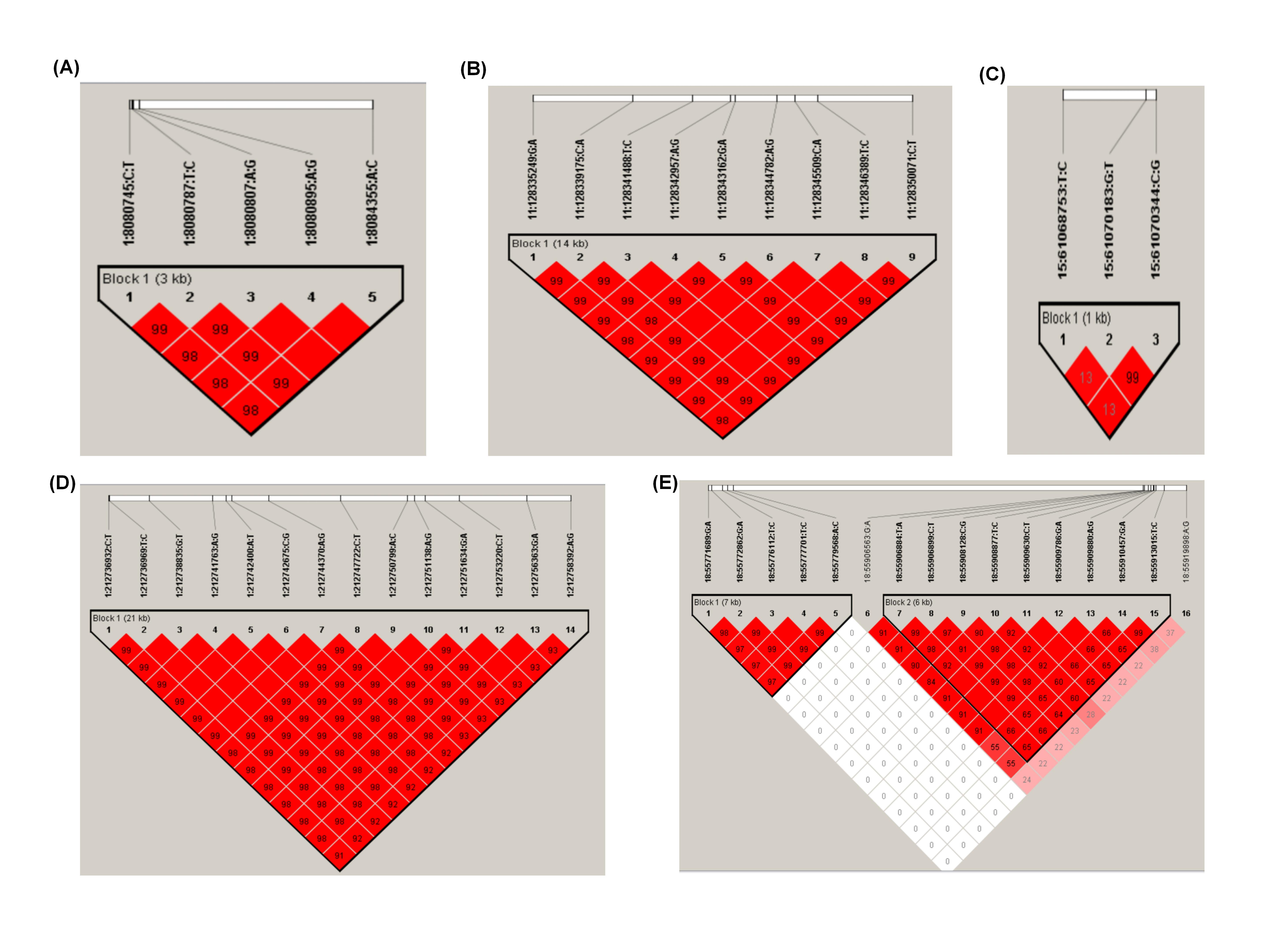


**SUPPLEMENTARY FIGURE 2:** Linkage disequilibrium (LD) analysis for 47 significant SNPs located in 5 genes. (A) 5 SNPs on *ERRFI1*, (B）9 SNPs on *ETS1,* (C)3 SNPs on *RORA,* (D) 14 SNPs on *ATF3,* (E) 16 SNPs on *NEDDL4.* SNPs, single nucleotide polymorphism; *ERRFI1,* ERBB Receptor Feedback Inhibitor 1; *ETS1,*v-ets avian erythroblastosis virus E26 oncogene homolog; *RORA*, retinoic acid receptor-related Orphan receptor alpha; *ATF3, Activating Transcription Factor 3; NEDD4L,* neural precursor cell expressed developmentally down-regulated 4-like.
