# Supplementary material for: Potentially functional variants of ERRFI1 in hypoxia‐related genes predict survival of non‐small cell lung cancer patients
Source: Cancer Med. 2024 Aug 3;13(15):e70073. doi: 10.1002/cam4.70073 (PMC11297539; doi:10.1002/cam4.70073)
Supplement: Supplementary file 2 — Figures S3–S6. [file CAM4-13-e70073-s001.doc]

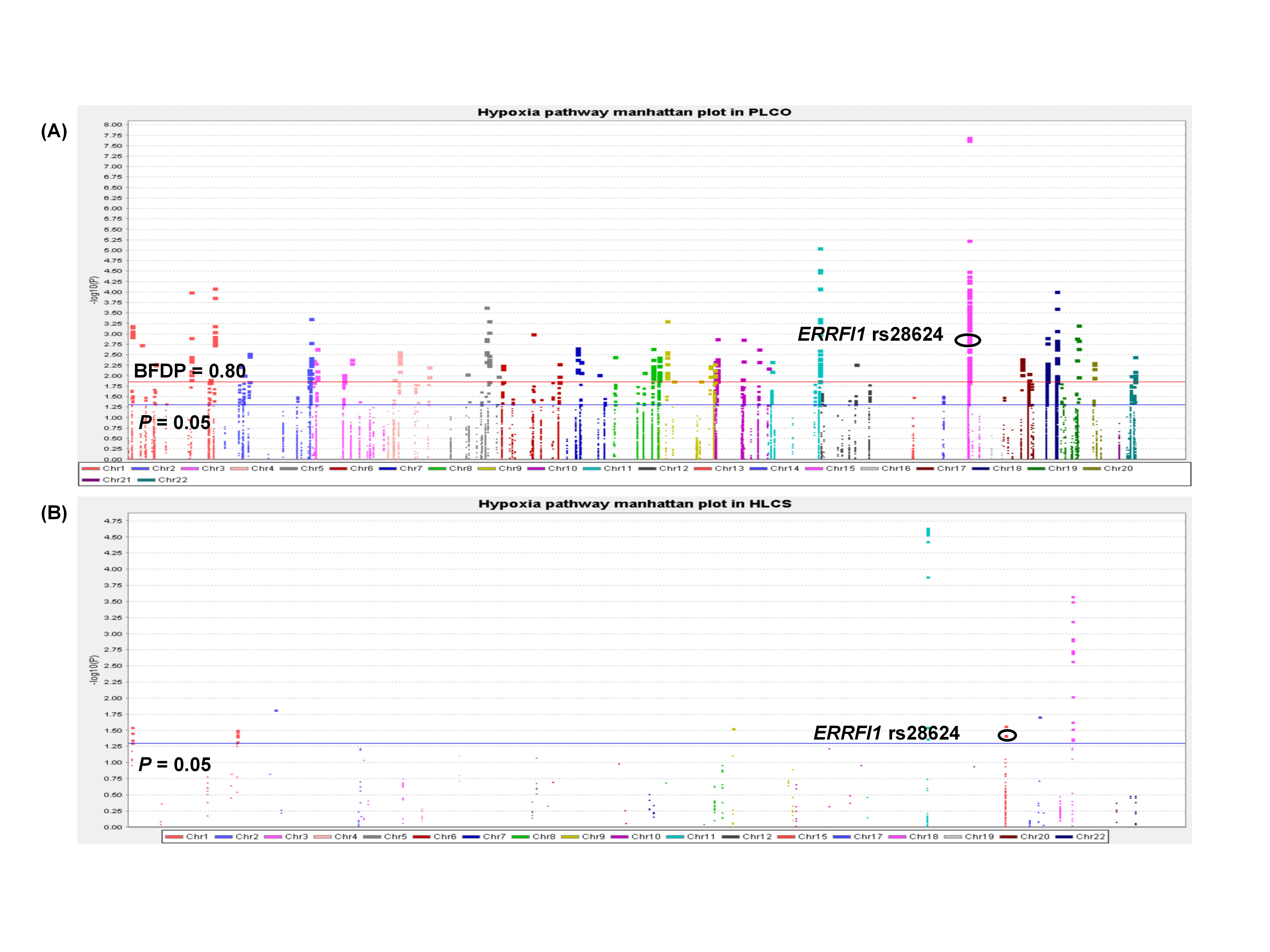


**SUPPLEMENTARY FIGURE 3.** Manhattan plot of association between genotype data and overall survival of patients with NSCLC in the PLCO trial and HLCS study (A)The statistical values across the autosomes of associations between 16,092 SNPs and overall survival in PLCO trial are plotted as −log10 *P* values. (B) The statistical values across the autosomes of associations between 630 SNPs and overall survival in HLCS study are plotted as −log10 *P* values. The blue horizontal line indicates *P* = 0.050 and the red line indicates BFDP = 0.800. NSCLC, non-small cell lung cancer; SNPs, single nucleotide polymorphism; PLCO, Prostate, Lung, Colorectal and Ovarian Cancer Screening trial; HLCS, Harvard Lung Cancer Suceptibility; OS, overall survival; BFDP, Bayesian false discovery probability; *ERRFI1,* ERBB Receptor Feedback Inhibitor 1.

**SUPPLEMENTARY FIGURE 4.** Regional association plots for the independent SNP in the hypoxia-related genes in the 1000 Genome Projects.SNPs in the region of 50 kilobases up or downstream of rs28624 in *ERRFI1* with 50 kb up-down-stream of the gene region,. Data points are colored according to the level of linkage disequilibrium of each pair of SNPs based on the hg19/1000 Genomes European population. The left-hand y-axis shows the association *P*-value of individual SNPs in the discovery dataset, which is plotted as -log10 (P) against chromosomal base-pair position. The right-hand y-axis shows the recombination rate estimated from HapMap Data Rel 22/phase II European population. SNPs, single nucleotide polymorphism; *ERRFI1,* ERBB Receptor Feedback Inhibitor 1*.*


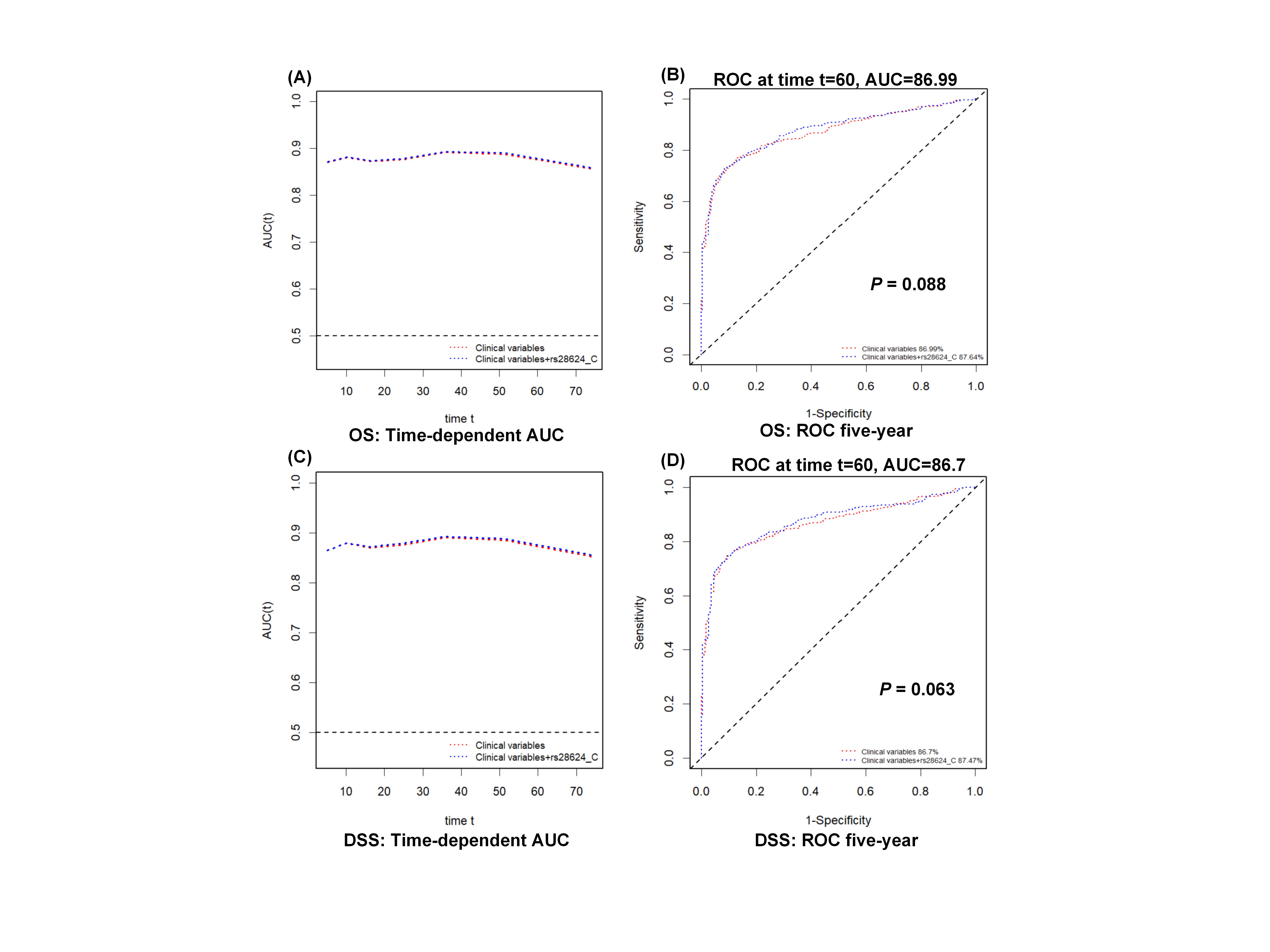


**SUPPLEMENTARY FIGURE 5.** Five-year NSCLC survival prediction of rs28624 by ROC curve in PLCO dataset. (A), (C) Time-dependent AUC estimation for OS and DSS: based on age, sex, smoking status, histology, tumor stage, chemotherapy, surgery, principal component and the two unfavourable genotypes, (B) Five-year NSCLC OS prediction by ROC curve, (D) Five-year NSCLC DSS prediction by ROC curve. NSCLC, non-small cell lung cancer; SNP, single-nucleotide polymorphism; ROC, receiver operating characteristic curve; AUC, area under curve; OS, overall survival; DSS, disease-specific survival; PLCO, The Prostate, Lung, Colorectal and Ovarian Cancer Screening Trial.


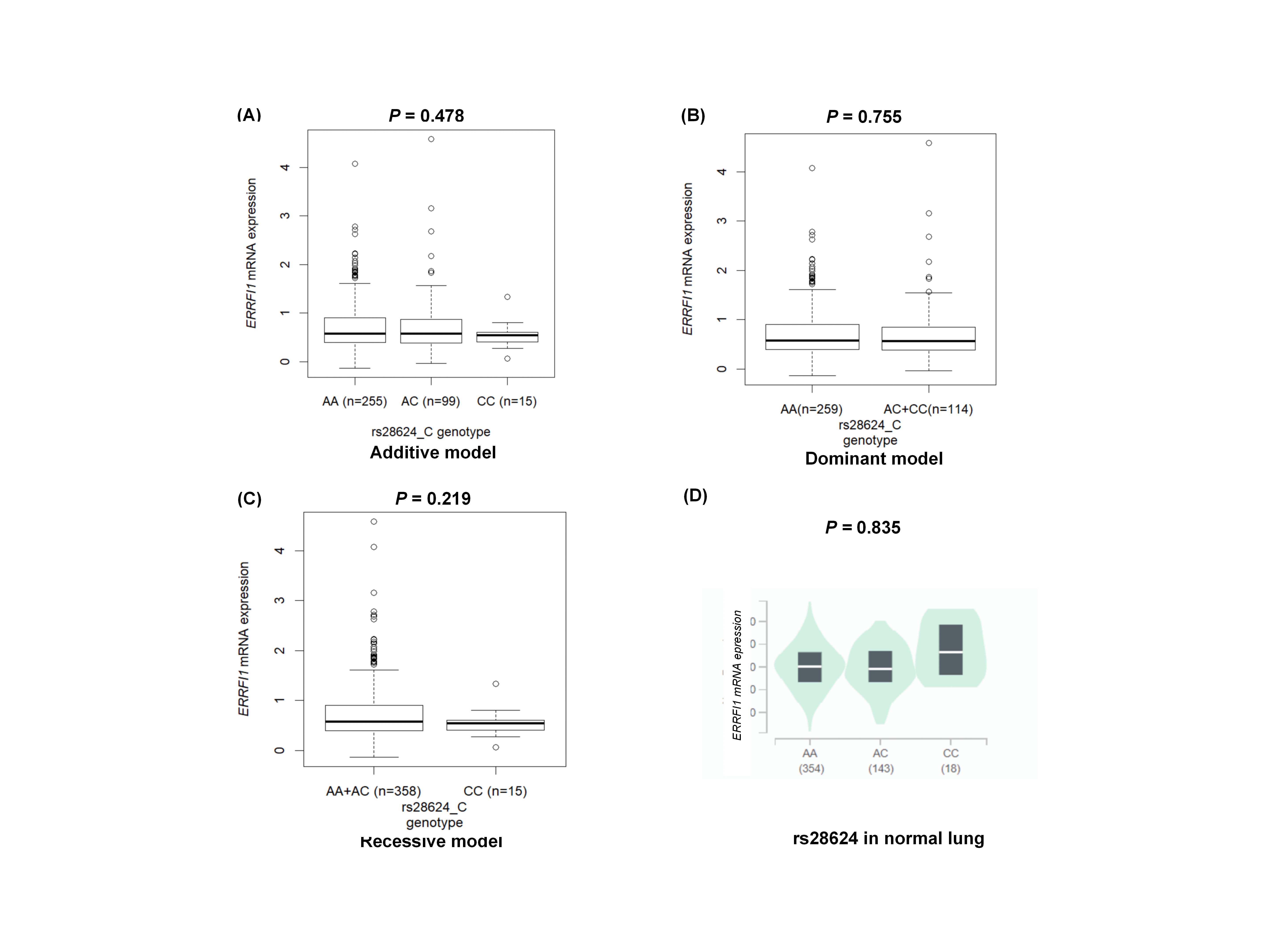


**SUPPLEMENTARY FIGURE 6**.Correlation of rs28624 genotypes with their corresponding mRNA expression levels.. The eQTL for *ERRFI1* rs28624. (A) in additive model, (B) dominant model and (C) recessive model from 1000 Genomes project; the eQTL for *ERRFI1* rs28624 (D) in normal lung from GTEx project. eQTL, expression quantitative trait loci; *ERRFI1,* ERBB Receptor Feedback Inhibitor 1.
