# Supplementary material for: Potentially functional variants of ERRFI1 in hypoxia‐related genes predict survival of non‐small cell lung cancer patients
Source: Cancer Med. 2024 Aug 3;13(15):e70073. doi: 10.1002/cam4.70073 (PMC11297539; doi:10.1002/cam4.70073)
Supplement: Supplementary file 3 — Figures S7–S8. [file CAM4-13-e70073-s003.doc]

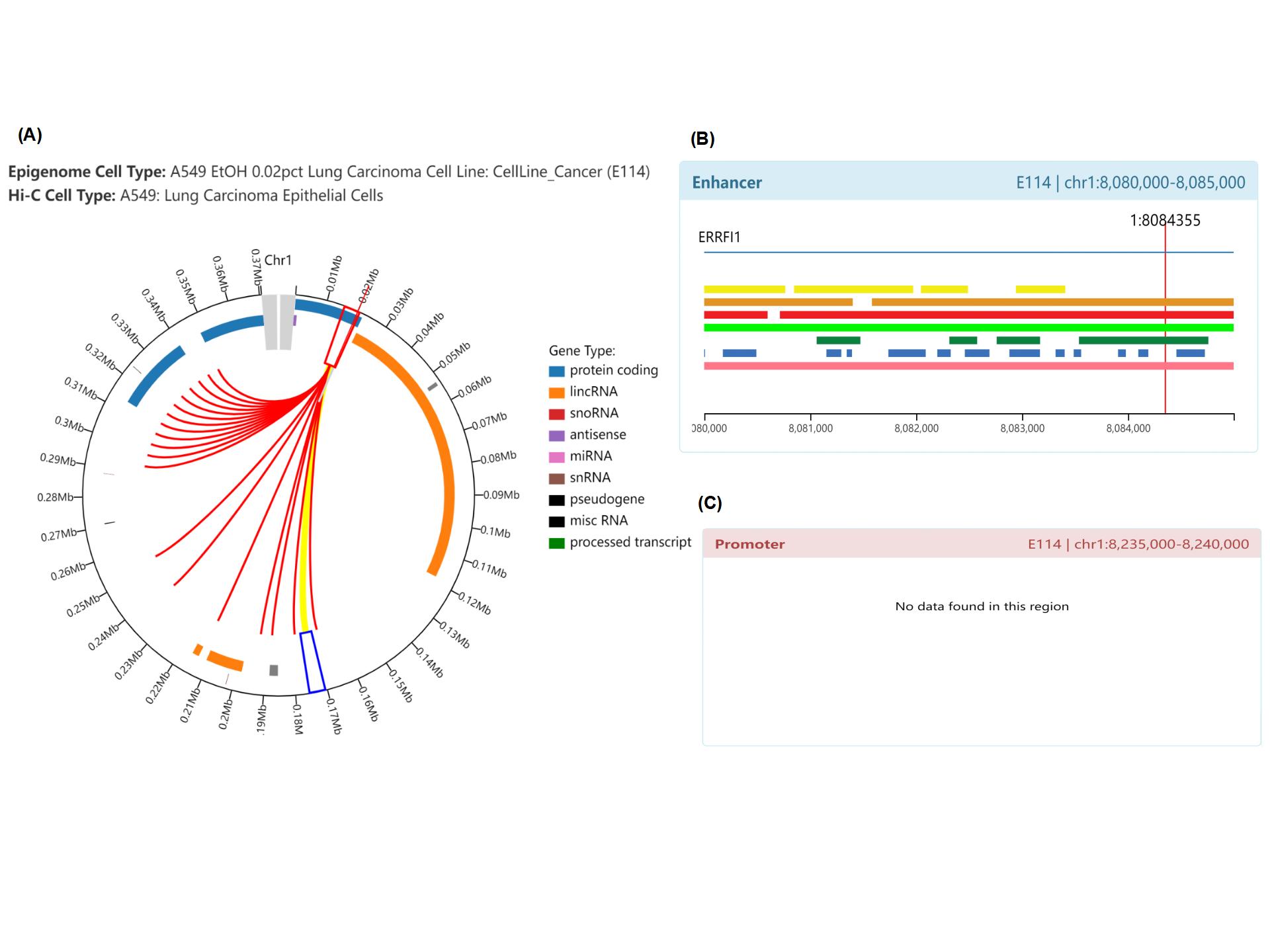


**SUPPLEMENTARY FIGURE 7**: In Silico Analysis of rs28624 and Its Regulatory Impact in A549 Lung Carcinoma Cells. (A) the genomic landscape and variant analysis of the rs28624 locus in A549 lung carcinoma cells, (B)Enhancer regions in the genomic vicinity of rs28624, including various gene types and their expression patterns. (C) Enhancer and promoter regions in the genomic vicinity of rs28624.


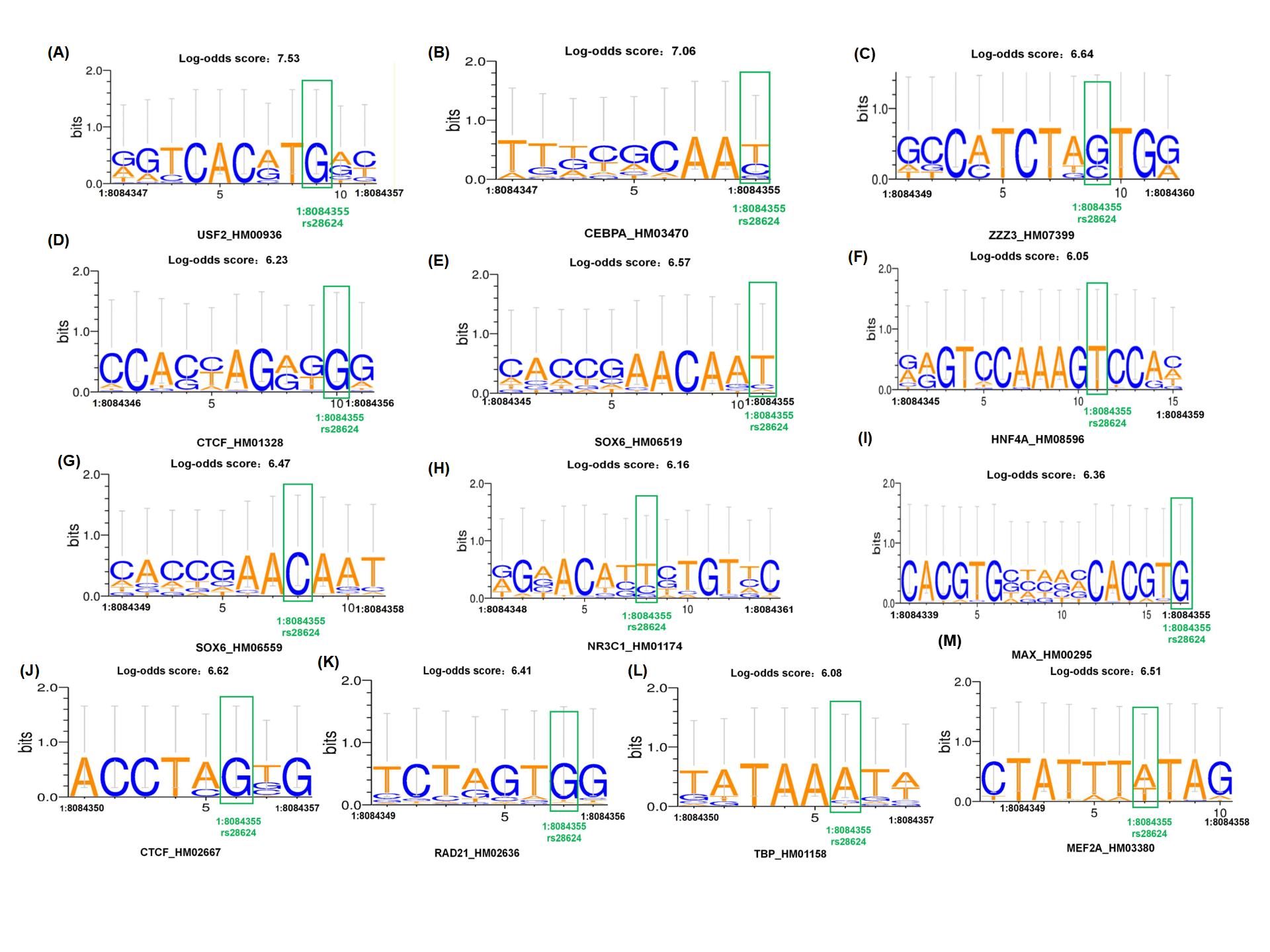


**SUPPLEMENTARY FIGURE 8**: Regulatory Motifs Affected by rs28624 Variant from the GWAS4D database (2018). Each subplot (A-M) represents a motif logo for a different transcription factor, showing the nucleotide sequence with the binding site and the frequency of each base at each position. (A) USF2_HM00936, log-odds score: 7.53, (B) CEBPA_HM03470, log-odds score: 7.06, (C) ZZZ3_HM07399, log-odds score: 6.64, (D) CTCF_HM01328, log-odds score: 6.23, (E) SOX6_HM06519, log-odds score: 6.57, (F) HNF4A_HM08596, log-odds score: 6.05, (G) SOX6_HM06559, log-odds score: 6.47, (H) NR3C1_HM01174, log-odds score: 6.16, (I) MAX_HM00295, log-odds score: 6.36, (J) CTCF_HM02667, log-odds score: 6.62, (K) RAD21_HM02636, log-odds score: 6.41, (L) TBP_HM01158, log-odds score: 6.08,(M) MEF2A_HM03380, log-odds score: 6.51.
